# A Cysteine-Rich Protein, SpDIR1L, Implicated in S-RNase-Independent Pollen Rejection in the Tomato (*Solanum* Section *Lycopersicon*) Clade

**DOI:** 10.3390/ijms222313067

**Published:** 2021-12-02

**Authors:** Juan Vicente Muñoz-Sanz, Alejandro Tovar-Méndez, Lu Lu, Ru Dai, Bruce McClure

**Affiliations:** 1 Department of Biochemistry, University of Missouri, Columbia, MO 65211, USA; alejandro@elementalenzymes.com (A.T.-M.); lul@health.missouri.edu (L.L.); rudai@ufl.edu (R.D.); mcclureb@missouri.edu (B.M.); 2 Rijk Zwaan Iberica S.A., Carretera Viator Paraje El Mamí S/N, La Cañada, 04120 Almería, Spain; 3 Elemental Enzymes, 1685 Galt Industrial Boulevard, St. Louis, MO 63132, USA; 4 Department of Medicine, University of Missouri School of Medicine, Columbia, MO 65211, USA; 5 Department of Horticultural Sciences, University of Florida, Fifield Hall, 2550 Hull Road, Gainesville, FL 32611, USA

**Keywords:** interspecific reproductive barriers, unilateral incompatibility, stylar factor, DIR1-like, *Solanum pennellii*, *Solanum lycopersicum*

## Abstract

Tomato clade species (*Solanum* sect. *Lycopersicon*) display multiple interspecific reproductive barriers (IRBs). Some IRBs conform to the SI x SC rule, which describes unilateral incompatibility (UI) where pollen from SC species is rejected on SI species’ pistils, but reciprocal pollinations are successful. However, SC x SC UI also exists, offering opportunities to identify factors that contribute to S-RNase-independent IRBs. For instance, SC *Solanum pennellii* LA0716 pistils only permit SC *Solanum lycopersicum* pollen tubes to penetrate to the top third of the pistil, while *S. pennellii* pollen penetrates to *S. lycopersicum* ovaries. We identified candidate *S. pennellii* LA0716 pistil barrier genes based on expression profiles and published results. CRISPR/Cas9 mutants were created in eight candidate genes, and mutants were assessed for changes in *S. lycopersicum* pollen tube growth. Mutants in a gene designated *Defective in Induced Resistance 1-like* (*SpDIR1L*), which encodes a small cysteine-rich protein, permitted *S. lycopersicum* pollen tubes to grow to the bottom third of the style. We show that SpDIR1L protein accumulation correlates with IRB strength and that species with weak or no IRBs toward *S. lycopersicum* pollen share a 150 bp deletion in the upstream region of *SpDIR1L*. These results suggest that *SpDIR1L* contributes to an *S*-RNase-independent IRB.

## 1. Introduction

Interspecific reproductive barriers (IRBs) are both biologically and practically significant. They are crucial for speciation, and instances of interspecific hybridization can also be important in plant evolution [1,2]. In crop breeding, IRBs may hinder access to wild germplasm [3,4]. Interspecific pollination is also very common in some natural contexts, and it may have a substantial fitness cost [5]. Pre-zygotic IRBs based on pollen–pistil interactions are of special interest, as they can block fertilization and, thus, mitigate the negative effects of interspecific pollination [5,6]. It is noteworthy that IRBs may result from evolutionary divergence between pollen and pistil, a phenomenon referred to as incongruity, as well as from incompatibility, which we regard as active recognition and rejection of interspecific pollen [7,8].

Although there are increasing numbers of mechanistic studies of IRBs, intraspecific self-incompatibility (SI) pollen rejection is better understood and provides a paradigm. SI species in Solanaceae display S-RNase-based gametophytic SI [7] characterized by pistil-expressed S-RNases and multiple pollen-expressed F-box protein genes encoded at the *S*-locus. S-RNase expression is under developmental control, rather than being a response to pollination, and, therefore, its cytotoxic activity presents a constitutive barrier to pollen that has no resistance mechanism [9,10,11]. The pollen-expressed *S*-locus F-box (SLF) proteins provide resistance. They are thought to be distributed such that SLF genes in a particular *S*-haplotype provide resistance to every possible S-RNase except the one encoded by the same *S*-haplotype [12,13,14]. Thus, in this system, a plant displays SI precisely because it cannot overcome the S-RNase barrier in its own pistil. Additional factors that do not contribute to *S*-specificity per se include HT-proteins [15], 120 kDa [16], NaTrxh [17], and NaStEP [18] on the pistil side; and NaSIPP [19] and SCF complex proteins such as Skp1-like [20] and Cullin1 on the pollen side [21]. A relationship between IRBs and S-RNase-based SI was first established in *Nicotiana*, but the relationship is complex, and multiple mechanisms with partially overlapping factor requirements clearly exist [22]. In *Nicotiana*, some S-RNase-based interspecific pollen rejection mechanisms require factors similar to those needed for SI, and others do not [22]. Moreover, some *Nicotiana* IRBs do not require S-RNase at all [22].

The tomato clade has advantages for elucidating both S-RNase-dependent and S-RNase-independent IRBs. It consists of 13 species: four self-compatible (SC) red/orange-fruited species (including *Solanum lycopersicum*) and nine green-fruited species, of which seven are predominantly SI and two are SC [23]. Tomato clade interspecific crossing relationships have been comprehensively characterized [24]. Most conform to the SI x SC rule: a common form of unilateral incompatibility (UI) where pollen from SI species is compatible on pistils of related SC species, but the reciprocal pollinations are rejected [7,25,26]. The prevalence of IRBs that conform to the SI x SC rule implies a relationship with SI because it is the SI pistil that rejects pollen, while the SC species pistil accepts. Experiments confirm this for crosses between red/orange-fruited SC species and green-fruited SI tomato clade species. For example, expressing functional *S-RNase* and *HT* genes from SI species causes rejection of pollen from red/orange-fruited SC species [10]. Similarly, an SLF protein and a CUL1 are required for pollen resistance to an S-RNase-dependent IRB [27,28].

The tomato clade also provides examples of SC x SC UI relationships that do not conform to the SI x SC rule. If only SI-related IRBs contribute to UI, then SC species should be cross-compatible, and this is often true [24]. However, there also are nonconforming examples where UI exists between SC tomato clade species. In cases where the species do not express functional S-RNase, these examples provide opportunities to elucidate S-RNase-independent IRBs. For instance, *S. lycopersicum* (tomato) displays UI with SC accessions of the predominantly SI species *S. arcanum*, *S. habrochaites,* and *S. pennellii* that fail to express active S-RNase [24,29,30,31]. Furthermore, quantitative studies of pollen tube growth after interspecific pollination provide evidence for multiple S-RNase-independent IRBs [24]. For example, rejection of *S. lycopersicum* pollen tubes in SC *S. pennellii* LA0716 pistils is quite different from rejection in SC *S. arcanum* LA2157. The former is described as ‘early’ rejection because *S. lycopersicum* pollen tubes only penetrate to the top part of the style, and the latter as ‘late’ rejection because they penetrate nearly to the base of the style [24]. Suppressing *HT-A* and *HT-B* expression in these accessions converts the ‘early’ rejector *S. pennellii* LA0716 to a ‘late’ type, but, in stark contrast, converts the ‘late’ rejector *S. arcanum* LA2157 to full compatibility with *S. lycopersicum*, thus allowing otherwise inaccessible hybrids to be recovered [32]. These results show that SC *S. pennellii* LA0716 and SC *S. arcanum* LA2157 both display S-RNase-independent IRBs that require HT-proteins, but SC *S. pennellii* LA0716 also possesses at least one additional barrier that is not present in *S. arcanum* LA2157. 

HT-genes are known to contribute to pollen rejection, albeit in SI, rather than the interspecific context. However, two recent studies identified IRB genes with no previously known role in pollen rejection. One study identified a pollen-side gene for another S-RNase-independent barrier in SC *S. pennellii* LA0716 [33]. Since pollen lacking resistance to a pistil-side barrier is rejected, the authors used transmission ratio distortion (TRD) to identify an *S. pennellii* LA0716 QTL contributing to UI between *S. pennellii* and *S. lycopersicum*. The underlying gene was identified as a farnesyl pyrophosphate synthase (*FPS2*). The *S. lycopersicum* allele shows 18-fold lower expression than the *S. pennellii* LA0716 allele, suggesting that pollen resistance is related to higher FPS expression. Recently, an *S. pennellii* ornithine decarboxylase gene, *ODC2*, has been implicated in the corresponding pistil-side barrier [34]. *ODC2* knockout mutants do not reject pollen with reduced farnesyl pyrophosphate synthase activity, and abolish the TRD just mentioned. *ODC2* has also been shown to interact with *ui12.1*, a locus involved in LA0716 *S. pennellii* LA0716 x *S. lycopersicum* UI that co-localizes with HT genes [31], strengthening *FPS2*-based pollen rejection.

Here, we evaluated eight candidate pistil-side barrier genes to better understand SC *S. pennellii* LA0716 x *S. lycopersicum* IRBs. Published RNASeq data [35] were used to identify five genes preferentially expressed in IRB-competent *S. pennellii* LA0716 pistils, and an additional three genes were selected because they had previously been implicated in pollen–pistil interactions in other species. Homozygous *S. pennellii* LA0716 CRISPR/Cas-9 knockout mutants were created in each gene and assessed for changes in *S. lycopersicum* pollen tube growth. Mutations in one gene, *Defective in Induced Resistance 1-like*, *SpDIR1L*, converted the ‘early’ *S. lycopersicum* pollen rejection phenotype characteristic of *S. pennellii* LA0716 to a ‘late’ phenotype. *SpDIR1L* encodes a small Cysteine-Rich Protein (CRP) expressed in mature *S. pennellii* LA0716 pistils but not in immature pistils or in mature *S. lycopersicum* pistils. Other tomato-clade species that display strong IRBs also accumulate SpDIR1L protein, but species with weakened IRBs do not. Species with weak IRBs share a 150 bp deletion upstream of the *SpDIR1L* start codon that could account for decreased expression. We conclude that *SpDIR1L* is implicated in an S-RNase-independent IRB between SC *S. lycopersicum* pollen by SC *S. pennellii* LA0716.

## 2. Results

### 2.1. Candidates and CRISPR/Cas9 Mutants

Both S-RNase-independent and S-RNase-dependent IRBs are developmentally controlled in *S. pennellii* LA0716 itself [11]. Briefly, pistil-side barriers are not present five days prior to anthesis, allowing SC *S. lycopersicum* pollen tubes to penetrate to the ovary at this stage (−5 stage). However, the pistil becomes competent for both IRB rejection of SC *S. lycopersicum* pollen tubes and, in SI accessions, self-pollen tubes, two to three days prior to anthesis (−2 to −3 stage, [11]).

**Figure 1:**
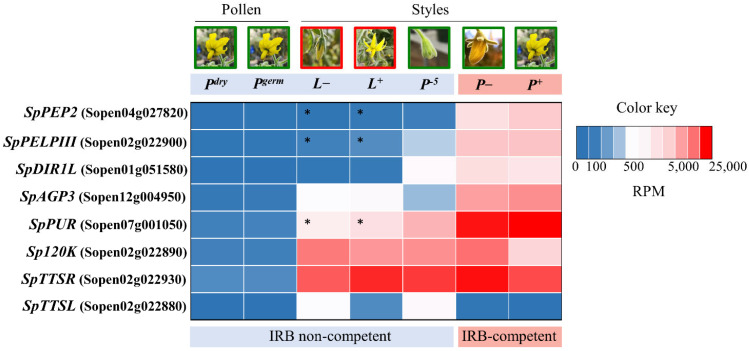
Heat map illustrating normalized expression levels. Published data from [35] were mapped to the *S. pennellii* genome, normalized, and presented in heat map form (red, high; blue, low) as shown. Columns show the tissues analyzed to characterize expression profile: *P^dry^*, *S. pennellii* LA0716 ungerminated (dry) pollen; *P^germ^*, *S. pennellii* LA0716 germinated pollen; *L^−^*, *S. lycopersicum* styles −1 stage, 1 day prior to anthesis; *L^+^*, *S. lycopersicum* styles, +1 stage, anthesis; *P^−5^*, *S. pennellii* LA0716 styles −5 stage, 5 days prior to anthesis; *P^−^*, *S. pennellii* LA0716 styles −1 stage, 1 day prior to anthesis; *P^+^*, *S. pennellii* LA0716 styles +1 stage, anthesis. IRB-competent and non-competent samples are indicated. Asterisks, loss of function mutations in *S. lycopersicum*.

We favored candidates with expression patterns consistent with this biological pattern and that display relatively high-level expression. In agreement with results from [35], we selected three *S. pennellii* LA0716 genes, Sopen04g027820, Sopen01g051580, and Sopen02g022900, for testing. Figure 1 compares expression of these and other candidates in IRB competent and non-competent samples. Sopen04g027820 encodes a pectin methylesterase inhibitor that we refer to as Pistil-Expressed Peptide 2 (SpPEP2). The corresponding *S. lycopersicum* gene could not be identified, as it appears to have been deleted from the genome. Sopen01g051580 encodes a small Cysteine-Rich Protein (CRP) similar to Defective in Induced Resistance 1-Like (SpDIR1L) of *A. thaliana*. Sopen02g022900 encodes a class III Pistil-specific Extension-Like Protein (SpPELPIII) similar to *Nicotiana tabacum* PELPIII, which has been implicated in an IRB in *Nicotiana* [36]. It is noteworthy that the corresponding *S. lycopersicum* sequence contains a nonsense mutation and is split into two gene models (Solyc02g078060 and Solyc02g078070). Two additional candidates were not among the top differentials identified by [35]. Sopen12g004950 shows a modest, but substantial (~25 fold), expression difference between mature *S. lycopersicum* and *S. pennellii* pistils (Figure 1). Nevertheless, its high level of expression and strong induction relative to the −5 stage resemble known pollination factors, such as *HT* and *S-RNase*. The encoded protein is a non-classical arabinogalactan-protein (AGP), referred to here as SpAGP3. It is similar to AGPNa3, a stigma-specific protein from *N. alata* [37]. Sopen07g001050 encodes a CRP (a purothionin, SpPUR), and it shows the second highest expression level in +1 stage *S. pennellii* LA0716 pistils, after the *HT-A* gene. A corresponding reading frame is present in *S. lycopersicum* beginning at position 2,380,292 on chromosome 7 (SL2.50), but it is not annotated because the ATG start codon is mutated to ATT. Thus, although the difference in transcript accumulation is modest, the SpPUR protein is likely not present in *S. lycopersicum*.

Three additional candidates, Sopen02g022880, Sopen02g022890, and Sopen02g022930, were chosen because orthologous genes are linked to pollen–pistil interactions. These three genes occur in a cluster with the SpPELPIII gene candidate, Sopen02g022900. Members of this four-gene cluster have similar intron-exon structures ([38]; Supplementary Figure S1, see Supplementary Materials), and the encoded proteins have conserved C-terminal Ole E1 domains and divergent proline-rich domains. Sopen02g022890, here referred to as *Sp120K*, is orthologous to the 120 kDa glycoprotein (120K) gene first characterized in *N. alata* [39,40] and implicated in SI [16]. Sopen02g022880 and Sopen02g022930, here referred to, respectively, as *SpTTSL* and *SpTTSR*, encode proteins similar to the transmitting-tract-specific (TTS) proteins first characterized in *N. tabacum* [41,42] and are thought to support compatible pollen tube growth. Sequence analyses across Solanaceae suggest that these genes are under positive selection and a role in speciation has been proposed [6]. Expression data for these genes are included in Figure 1. Like the other candidates, *Sp120K* and *SpTTSR* are expressed at extraordinary levels in mature SC *S. pennellii* LA0716 pistils. As mentioned by other authors [6], *SpTTSL* expression is neither as specific to the pistil nor is it as highly expressed as *SpTTSR* orthologs, but it was nevertheless included because of its similar structure.

Mutations were created in each candidate gene using CRISPR/Cas9. Gene regions close to the 5′ end were targeted, to the extent possible. Table 1 shows the targeted mutation positions relative to the mature ORFs, and Figure S1 shows the sequence contexts and other details. T_0_ plants were screened for frameshifting indels [43], and the mutated segments were sequenced. Most mutants were small indels, but large indels and biallelic mutations (i.e., a different mutation in each allele) were also recovered. For each of the eight genes, T_0_ mutants were selfed, and up to three homozygous lines were identified for testing pollination phenotypes. Table 1 and Figure S1 show the frameshifting indel alleles (named as *spagp3-1*, *spagp3-2*, *spagp3-3*, etc.) and the positions of the introduced premature stop codons. Altogether, 18 mutant alleles, including one to three lines homozygous for mutations in each candidate gene, were assessed for changes in *S. lycopersicum* pollen tube growth.

### 2.2. SpDIR1L Behaves as an IRB Barrier Gene

**Figure 2:**
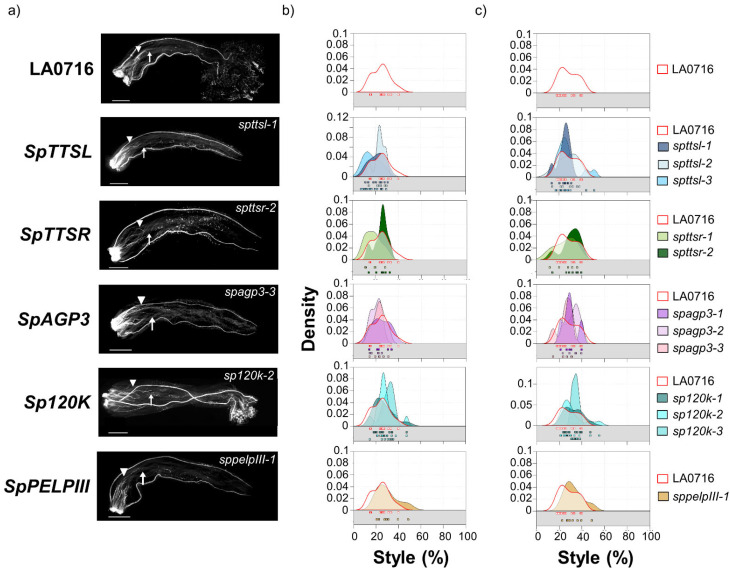
IRB phenotypes of loss-of-function of CRISPR/Cas9 mutations that showed no significant difference from control. (**a**) *S. lycopersicum* pollen tubes in control (top row) and in five *S. pennellii* LA0716 candidate genes (*SpTTSL*, *SpTTSR*, *SpAGP3*, *Sp120K,* and *SpPELPIII*). Homozygous mutants were emasculated, pollinated with *S. lycopersicum* pollen the next day, and then imaged after 48 h. The specific mutant alleles are shown. Triangles, location where most pollen tubes stopped; arrows, longest visible pollen tube; bar, 1 mm. (**b**,**c**): Summary kernel density plots showing where most pollen tubes (**b**) or longest visible pollen tubes (**c**) were arrested, expressed as percent of style traversed (x-axis). Untransformed control data are shown in each plot to facilitate direct comparison. Squares below each kernel density plot show the pollination used to plot kernel distributions.

**Figure 3:**
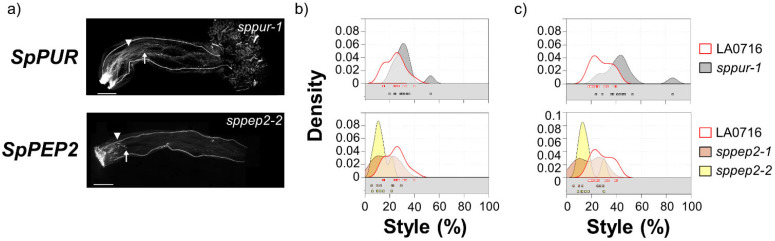
IRB phenotypes of loss-of-function of CRISPR/Cas9 mutations in mutants that showed significant differences from controls but do not support roles as IRB gene barriers. (**a**) *S. lycopersicum* pollen tubes in two *S. pennellii* LA0716 candidate genes, *SpPUR,* and *SpPEP2*. Homozygous mutants were emasculated, pollinated with *S. lycopersicum* pollen the next day, and then imaged after 48 h. The specific mutant alleles are shown. Triangles, location where most of pollen tubes stopped; arrows, longest visible pollen tube; bar, 1 mm. (**b**,**c**): Summary kernel density plots showing where most pollen tubes (**b**) or longest visible pollen tubes (**c**) were arrested, expressed as percent of style traversed (x-axis). Untransformed control data are shown in each plot to facilitate direct comparison. Squares below each kernel density plot show the pollination used to plot kernel distributions.

**Figure 4:**
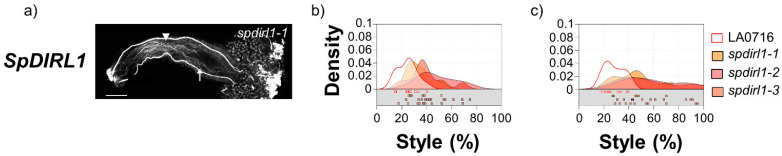
IRB phenotypes of loss-of-function of CRISPR/Cas9 mutations that showed significant differences from control. (**a**) *S. lycopersicum* pollen tubes in *S. pennellii* LA0716 candidate gene *SpDIR1L*. Homozygous mutants were emasculated, pollinated with *S. lycopersicum* pollen the next day, and then imaged after 48 h. The specific mutant allele is shown. Triangle, location where most pollen tubes stopped; arrow, longest visible pollen tube; bar, 1 mm. (**b**,**c**): Summary kernel density plots showing where most pollen tubes (**b**) or longest visible pollen tubes (**c**) were arrested, expressed as percent of style traversed (x-axis). Untransformed control data are shown in each plot to facilitate direct comparison. Squares below each kernel density plot show the pollination used to plot kernel distributions.

Figure 2, Figure 3 and Figure 4 summarize interspecific pollination results for loss-of-function mutants in the eight candidate genes. *S. pennellii* pistils were emasculated before anthesis (−1 stage, [11]), and heavily pollinated with *S. lycopersicum* pollen the next day (+1), and pollen tube rejection was assessed by aniline blue fluorochrome staining 48 h later. Sample pollen tube images are shown on the left (Figure 2a, Figure 3a, and Figure 4a). Pollen tube rejection is always asynchronous, so two metrics were to describe the IRBs, and the plotted data show the dispersion as well. The point where most pollen tubes have stopped (no more than five pollen tubes passed; Figure 2b, Figure 3b, and Figure 4b) reflects the overall response of the majority of *S. lycopersicum* pollen tubes. However, single pollen tubes are also relevant, so the lengths of the longest visible pollen tubes are plotted separately (Figure 2c, Figure 3c, and Figure 4c). Both metrics are presented as percentages of style length. Data for untransformed control pistils (top) are shown in each plot to facilitate comparison. Individual data points (boxes) are also shown. In untransformed controls, most *S. lycopersicum* pollen tubes grew no more than 25% of the style length (mean ± SD = 24.0 ± 7.8%, range 14–39%, Figure 2a,b; Supplementary Table S1. Mutant phenotypes for five candidates, all encoding glycoproteins, showed no significant difference from controls (Kruskal–Wallis, *p* < 0.05) as reflected by the positions where most *S. lycopersicum* pollen tubes stopped: *SpTTSL* (19.7 ± 7.0%, range 8–30%, three alleles), *SpTTSR* (22.0 ± 7.1%, range 11–33%, two alleles), *SpAGP3* (22.5 ± 5.9%, range 14–34%, three alleles), *Sp120K* (28.4 ± 7.5%, range 14–48%, two alleles), *SpPELPIII* (29.4 ± 9.8%, range 20–49%, one allele), and *SpPUR-1* (20–53%, 31.3 ± 8.6%, one allele). The longest pollen tube metrics for these genes show the same negative results (Table S2).

*SpPUR* and *SpPEP2* mutant phenotypes are noteworthy, but the results do not clearly support roles as IRB barrier genes. A small number of *S. lycopersicum* pollen tubes penetrated deeper into the style in the *sppur-1* mutant. This exaggerated the bimodal distribution (Figure 3c), and while the difference from control is significant by this measure (*p* = 0.002), the effect is modest in comparison to the overall population of *S. lycopersicum* pollen tubes. Styles in both *SpPEP2* mutants were notably shorter than controls (14.7 ± 7.5% vs. 24.0 ± 7.8% for control, Figure 3), and, by either measure, *S. lycopersicum* pollen tubes traversed a smaller percentage of the pistil than controls. The differences are significant (Kruskal–Wallis tests *p* = 0.003 and 0.005, for most or longest pollen tubes, respectively; Supplementary Table S2) and may implicate *SpPEP2* in pollen pistil interactions but not in UI between *S. pennellii* LA0716 and *S. lycopersicum*, as the direction of the effect is opposite from the behavior of a mutation in an IRB barrier gene.

In contrast, mutations in *SpDIR1L* resulted in increased penetration by *S. lycopersicum* pollen tubes (Figure 4a,b). Across three *dir1l* alleles, *S. lycopersicum* pollen tubes traversed an average of up to 41.61 ± 14.4% of the style, while the longest reached 54.2 ± 21.1%. This is almost half again as far as untransformed controls (24 or 28% for most, or longest, pollen tubes, respectively; Kruskal–Wallis *p* < 0.001; Supplementary Tables S1 and S2). The data dispersion in these mutants is also noteworthy. The maximum penetration for most *S. lycopersicum* pollen tubes ranged from 17 to 75% of the style, while the longest pollen tubes were distributed over the lower third of the style. Remarkably, in some crosses, the longest *S. lycopersicum* pollen tubes penetrated 90–95% of the style (Figure 4a,c); however, they never reached the ovary. Thus, *SpDIR1L* loss-of-function in *S. pennellii* LA0716 partially mitigates *S. lycopersicum* pollen tube rejection, but it does not completely abolish IRBs. This is expected because *S. pennellii* LA0716 displays at least two, and probably more, independent IRBs with additive effects [32].

### 2.3. DIR1L Genes Are Expressed in Species with Strong IRBs

**Figure 5:**
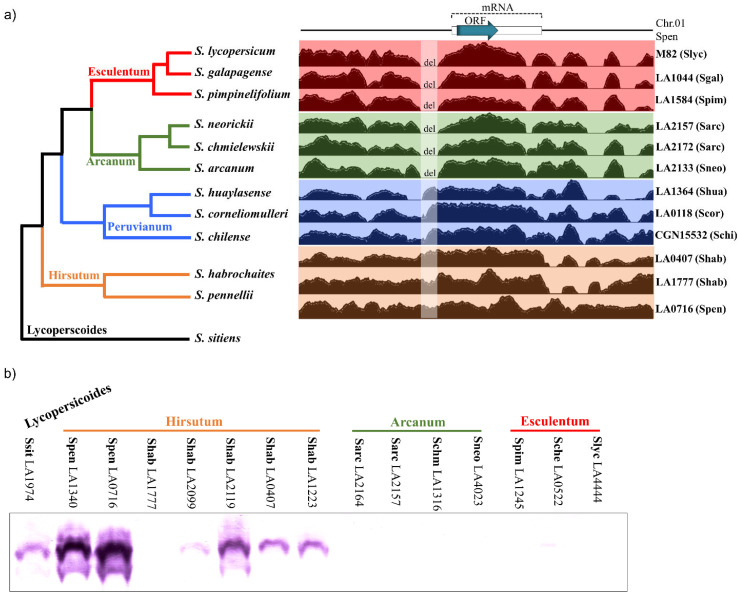
*DIR1L* expression across the tomato clade. (**a**) Left, simplified tomato clade phylogeny showing Esculentum, Arcanum, Peruvianum, and Hirsutum subgroups. *Solanum sitiens* (black branch) is included as an outgroup. Tree structure and branch lengths are based on [44,45]. Right, short read genomic data from representative accessions of each subgroup mapped onto the *S. pennellii* LA0716 genome. Read density in the region 1200 bp on either side of *SpDIR1L* is shown. Blue arrow, *SpDIR1L* ORF; white rectangle, predicted transcript; del, absence of reads from Esculentum and Arcanum species indicates a deletion shared by these species. (**b**) DIR1L protein accumulation. Accession numbers are given, species names abbreviated: Slyc = *S. lycopersicum,* Sgal = *S. galapagense,* Sche = *S. cheesmaniae,* Spim = *S. pimpinellifolium,* Sneo = *S. neorickii,* Schm = *S. chmielewskii,* Sarc = *S. arcanum,* Scor = *S. corneliomulleri,* Schi = *S. chilense,* Shab = *S. habrochaites,* Spen = *S. pennellii,* Ssit = *S. sitiens*.

Genomic data and protein blot analyses suggest a broad correlation between *DIR1L* gene expression and IRB strength in the tomato clade. Figure 5a (left) shows a simplified tomato clade phylogeny. The clade is partitioned into four major groups: Esculentum (including *S. lycopersicum*), Arcanum, Peruvianum, and Hirsutum (including *S. pennellii*) [23,44]. In general, the strongest pistil-side IRBs occur in the Hirsutum group, lesser barrier strength is observed in the Arcanum group, and species in the Esculentum group are cross-compatible [24]. Recent results suggest increasing barrier strength is due to multiple barriers with additive effects as opposed to single more potent barriers [8,9,32].

We examined *DIR1L* genes across the tomato clade to better understand how they might contribute to overall IRB strength. Figure 5a (right) shows results of mapping short read genomic sequence data onto a 2714 bp region including 1200 bp on either side of the *S. pennellii* LA0716 SpDIR1L coding region. For this analysis, each of the four major tomato clade groups is represented by three species. Remarkably, all six Esculentum and Arcanum group species display a 150 bp deletion beginning 127 bp upstream of the SpDIR1L coding region (75 bp upstream of the predicted transcription start site). Other indels were detected that do not correlate with tomato clade phylogeny (Figure 5a), although three SNPs were consistently present in Esculentum and Arcanum species (107,161,032 (A > C), 107,162,006 (G > A), and 107,162,194 (C > A); *S. pennellii* genome version [46]). The predicted DIR1L proteins across all twelve species showed 93–100% identity, but no frame-shifting mutations were detected.

Since the RNASeq data showed a >1000-fold SpDIR1L signal difference between *S. lycopersicum* and *S. pennellii LA0716* [35], we hypothesized that this deletion would be reflected at the protein level. To test this, we prepared an antipeptide SpDIR1L antibody to probe pistil extracts. Figure 5b confirms that SpDIR1L proteins are not detectable in pistil extracts from Esculentum and Arcanum group species that share the 150 bp deletion but are detectable in the Hirsutum group and an outgroup, *S. sitiens*. The Esculentum group is represented by *S. lycopersicum* LA4444 as well as *S. cheesmaniae* LA0522 and *S. pimpinellifolium* LA1245, two SC species that are cross compatible with *S. lycopersicum*. The Arcanum group is represented by the two SC species, *S. neorickii* LA4023 and *S. chmielewskii* LA1316, and two *S. arcanum* accessions (SI LA2164 and SC LA2157). Results for a selection of SI and SC accessions of Hirsutum group species are shown. Interestingly, SpDIR1L protein accumulation in SI *S. habrochaites* LA1777 is low and not visible in Figure 5b, but four other accessions (LA2099, LA2119, LA0407, and LA1223; all SC) and both *S. pennellii* accessions analyzed (LA1340 and LA0716, SI and SC, respectively) show robust accumulation.

## 3. Discussion

IRBs are more mechanistically diverse than the better-studied intraspecific SI systems. Most IRBs characterized so far are active systems in the sense that specific barrier genes are expressed in the pistil, and thus compatible conspecific pollen must express appropriate resistance. We refer to this as a barrier/resistance architecture [8,9,10]. This term may describe either SI or IRBs, and the intraspecific S-RNase-based SI system is, in fact, the best understood example wherein S-RNase and other factors comprise a pistil barrier and SLF and other factors provide for pollen resistance [8,9,47]. The barrier/resistance architecture can readily explain how UI arises. For example, in the context of interspecific crosses, UI will occur whenever there is a mismatch between pistil barriers in one species and pollen resistance in another. Thus, many species with S-RNase-based SI display interspecific UI with SC relatives because the latter have lost pollen resistance to the S-RNase barrier [8,10,27]. This contributes to the prevalence of interspecific UI relationships that conform to the SI x SC rule [8].

By extension, any pollen rejection mechanism that conforms to the barrier resistance architecture can cause UI if there is a mismatch between pistil barriers and pollen resistance. Thus, SC x SC UI systems offer the possibility of identifying new IRBs, beyond those linked to SI. Furthermore, the overall pattern of interspecific compatibility in the tomato clade is that species display greater or lesser degrees of IRB barrier strength on the pistil side [24]. SC *S. pennellii* LA0716 is notable for its strong IRBs, and SC *S. lycopersicum* pollen tubes are inhibited near the top of the pistil (i.e., early rejection phenotype). A confounding detail is that loss-of-function studies in *S. pennellii* LA0716 and other accessions (mainly in the Hirsutum and Peruvianum groups, Figure 5) suggest that greater IRB strength results from the additive effects of multiple barriers [32]. Thus, when multiple barriers are present, loss-of-function in one mechanism converts an early rejection phenotype to late rejection (i.e., inhibition near the stigma to inhibition near the base). In spite of these complications, our experiments focused on UI between *S. pennellii* LA0716 x *S. lycopersicum* because the rejection response is robust, and the plant materials are well characterized.

In this study, we sought to identify pistil-expressed barrier genes to better understand the S-RNase-independent IRBs. We used the developmental profile of IRBs in *S. pennellii* LA0716 as one means to identify candidate barrier genes. Chalivendra et al. [11] showed that *S. pennellii* IRBs toward *S. lycopersicum* pollen share a similar developmental onset with SI, meaning that the pistil is permissive at early stages in development and both inter- and intra-specific barriers appear at later stages. Thus, immature pistils support pollen germination and tube growth, and new factors contributing to both SI and interspecific barriers are added in a gain-of-function manner toward maturity. In the interspecific context, adding a similar *S. lycopersicum* vs. *S. pennellii* (i.e., IRB non-competent vs. competent) comparison is appropriate [15,35,48]. We tested five candidates that broadly conform to this paradigm (*SpPEP2*, *SpPELPIII*, *SpDIR1L*, *SpAGP3,* and *SpPUR*, Figure 1). There are obvious mutations in the *S. lycopersicum* orthologs of some of these genes (SL2.5, Solgenomics). The *S. lycopersicum* ortholog of *SpPEP2* is deleted. The *S. lycopersicum* ortholog of *SpPELPIII* has a nonsense mutation, and its CDS are included in two gene models, Solyc02g078060 and Solyc078070. Similarly, there is no annotated gene model corresponding to the *S. lycopersicum* ortholog of *SpPUR* because the start codon is mutated to ATT, although there is substantial transcript accumulation (Figure 1). Three additional candidates (*Sp120K*, *SpTTSR*, and *SpTTSL*) were tested because orthologs in other species have been implicated in pollen–pistil interactions, and they occur in an intriguing gene cluster with *SpPELPIII*. Since *S. pennellii* pistils attain compatibility toward *S. lycopersicum* pollen at an early stage and only become competent for rejection near maturity [11], CRISPR/Cas9 barrier gene mutants should be more permissive than controls. Moreover, because multiple IRBs are active in *S. pennellii* LA0716, single gene mutations should increase compatibility toward *S. lycopersicum* pollen, but are unlikely to permit complete compatibility [32].

Results for *SpPEP2*, *SpAGP3*, *SpPUR*, *SpTTSL*, *SpTTSR*, *Sp120K,* and *SpPELPIII* do not support major roles as IRB genes, at least not for *S. lycopersicum* pollen rejection. Compared to controls, a small number of *S. lycopersicum* pollen tubes penetrated farther into the style in the *SpPUR* mutant, but the majority of pollen tubes appeared unaffected. *S. lycopersicum* pollen tubes were actually shorter in *SpPEP2* mutants than in controls, which is the opposite result expected from an IRB barrier gene. *SpPEP2* encodes a pectin methylesterase inhibitor, so it is plausible that it has a role in pollen–pistil interactions [49] and pectin methylesterases have been implicated in inter-strain cross compatibility in maize [50,51,52]. The decreased penetration by *S. lycopersicum* pollen tubes in *SpPEP2* mutants might suggest a role in supporting pollen tube growth (Figure 3). This role could potentially be tested with a gain-of-function experiment, but the effect is modest, and it cannot be an essential gene since it is not present in *S. lycopersicum*. It is somewhat surprising that none of the AGP genes affected *S. lycopersicum* pollen tube growth. *SpTTSL* is not as highly expressed as the other genes and is expressed in nonsexual organs, but orthologs of each of the others have been implicated in various pollen-pistil interactions: NaAGP3 and the 120KDa glycoprotein from *N. alata* are S-RNase binding proteins [16,53,54]; TTS from *N. tabacum* supports pollen tube growth [41,42], and a role speciation has been proposed [6]; and PELPIII from *N. tabacum* is implicated in UI with *N. obtusifolia* and *N. repanda* [36]. The expression profiles of the *S. pennellii* orthologs of all these genes are consistent with those in *Nicotiana* (Figure 1), but our results show no effects on *S. lycopersicum* pollen tubes (Figure 2). Perhaps, there is redundancy, and mutations in multiple genes would show different results, but previous studies have tested effects of single genes (e.g., [16]). In addition, IRBs will, of course, affect species differently, and our experiments only tested for effects in the *S. pennellii* LA0716 x *S. lycopersicum* system, so effects on other species cannot be excluded.

Of the eight genes tested, only *SpDIR1L* has the characteristics of a *S. pennellii* LA0716 x *S. lycopersicum* barrier gene. Its sequence is similar to *DIR1* from *Arabidopsis thaliana,* which encodes a lipid transport protein (LTP2) thought to carry the systemic acquired resistance signal to distant leaves [55]. Moreover, the physical characteristics of the mature SpDIR1L protein (78 residues, 8 cysteines, pI = 7.5) are similar to other small proteins implicated in pollen–pistil signaling [56,57]. We present results for three independent *SpDIR1L* loss-of-function mutants (Figure S1b). The sgRNA directed CRISPR/Cas9 cleavage just after the GAT codon encoding Asp-28, predicted to be the second residue in the mature protein (i.e., after cleavage of the 26-residue signal peptide). The three frameshift mutations could produce truncated 33 to 36 residue polypeptides compared to the wild-type 104 residue SpDIR1L protein (Supplementary Figure S1). *SpDIR1L* mutations permitted most *S. lycopersicum* pollen tubes to progress nearly 50% farther into the style than controls (42% vs. 24% for mutant and control, respectively, Figure 4b). The phenotype reflected by the longest pollen tube metric is more dramatic. *S. lycopersicum* pollen tubes are present in the lower third of the style and sometimes reach 95% of the style length (Figure 4c). This metric was never observed in untransformed controls. Therefore, we conclude that *SpDIR1L* contributes to *S. lycopersicum* pollen rejection. As expected, this effect is quantitative and the mutations result in a more permissive *S. pennellii* pistil, but not compatibility per se.

SpDIR1L proteins do not accumulate at significant levels in other species with weaker IRBs. The protein encoded by the *S. lycopersicum* ortholog, Solyc01g109390, is very similar to *SpDIR1L*, and the region targeted by our antipeptide antibody is identical. Nevertheless, DIR1L protein is undetectable in *S. lycopersicum* pistil extracts (Figure 5b). Given the magnitude of the difference in RNASeq signal (Figure 1), we hypothesized that transcription is affected. We analyzed data from *S. lycopersicoides* and representatives of each of the tomato clade species groups and found a deletion 127 bp upstream of the coding regions in the Esculentum and Arcanum groups (Figure 5a). As these groups have weak IRBs toward *S. lycopersicum*, we also tested for protein accumulation and found little or no DIR1L protein (Figure 5b). Apart from SI *S. habrochaites* LA1777, which showed little or no DIR1L protein, Hirsutum group species express abundant protein. Other studies have also found IRB differences among *S. habrochaites* accessions [58]. Together, the results are consistent with loss of *DIR1L* function in the common ancestor of the Esculentum and Arcanum groups, and we infer that this contributes to the overall weaker IRBs in these groups.

Our results suggest that DIR1L proteins alone are not sufficient for rejection of *S. lycopersicum* pollen. For example, *S. habrochaites* LA1223 expresses a DIR1L protein, but it accepts pollen from *S. lycopersicum* and other Esculentum group species (Figure 4b, [58]). In addition, [59] created *S. pennellii* LA0716 introgression lines (ILs), at least one of which, IL1-4, includes *SpDIR1L*. Notably, *S. habrochaites* LA1223 is the sole *S. habrochaites* accession that lacks a functional *HT-A* gene [58], and IL1-4 may be similar because both *S. lycopersicum HT*-genes are nonfunctional [60]. As *HT*-genes are implicated in some S-RNase-independent IRBs [32], these observations are suggestive. Epistasis between *SpDIR1L* and *HT-A* could be tested in a future gain-of-function experiment in *S. lycopersicum.* In addition to an HT-dependent IRB, *S. pennellii* LA0716 contains another unlinked IRB mechanism, so SpDIR1L epistasis could be associated with a still unknown factor(s) necessary to mount an HT-independent IRB. Presently, too many questions remain unanswered to propose a satisfying biochemical role for DIR1L. Further research is needed to elucidate the mechanism of DIR1L-dependent pollen rejection.

## 4. Materials and Methods

### 4.1. Plant Materials

Plant Materials were obtained from the C. M. Rick Tomato Genetics Resource Center (http://tgrc.ucdavis.edu accessed on 2 February 2015). Plants were grown in ProMixBX in greenhouses in Columbia, MO, USA. Natural light was supplemented to a 16 h light/8 h darkness photoperiod.

### 4.2. Candidate Gene Selection Criteria

Candidate selections were informed by transcriptome data, comparisons of individual SC *S. lycopersicum* vs. *S. pennellii* LA0716 genes, and literature about genes implicated in pollen–pistil interactions. We prioritized RNASeq transcriptome data for its ability to highlight genes showing comparatively high and specific expression in *S. pennellii* LA0716 vs. *S. lycopersicum* pistils.

*S. pennellii* LA0716 and *S. lycopersicum* expression data were obtained from Pease et al. [35]. RNASeq data were reanalyzed to identify a practical number of candidates as well as capture plausible candidates from the literature. As our focus was on pistil-side factors, we compared normalized expression levels in IRB-competent pistils (*S. pennellii* LA0716 pistils at 1 day prior to anthesis and at maturity) to IRB non-competent pistils and pollen (*S. lycopersicum* styles 1 day prior to anthesis, at anthesis, *S. pennellii* LA0716 styles 5 days prior to anthesis, and dry or germinated pollen). Since tomato clade IRBs show a developmental profile similar to SI [11], we favored candidates that are highly expressed, as are many SI-related genes. As discussed in the text, additional candidates were selected because ortholog had been implicated in pollen–pistil interactions in other systems.

### 4.3. CRISPR/Cas9 Constructs

Knockout mutants were generated by CRISPR/Cas9 using the pRLG108 vector kindly supplied by Tomáš Čermák and Daniel Voytas, University of Minnesota. This vector includes the eCAS9 nuclease and encodes Csy4 for guide RNA maturation. Targets were selected [61,62] in 5′ coding regions (Supplementary Figure S1). Potential off-target sites were screened using BLAST [63] searches against *S. pennellii* and *S. lycopersicum* genomes, and targets with more than 15 out of 18 bp identity were rejected. DNA fragments flanked by BsaI and SapI were synthesized as shown in Supplementary Table S3 (gBlocks, IDT), cloned into pGEM-T easy (Promega, Fitchburg, WI, USA) and assembled in pRLG108. Constructs were sequence verified before transformation.

### 4.4. Plant Transformation and Mutual Identification

*S. pennellii* LA0716 transformation was performed as described in [32], except that ammonium glufosinate (5.2 µg/mL) was the selectable marker. A PCR-based protocol [43] was used to detect indels in T_0_ transgenic plants (primer sets are given in Supplementary Table S4). Primers located outside the protospacer region (Supplementary Table S4) were used to amplify and clone mutated sequences from T_0_ plants. Six independent clones were sequenced to verify the indels.

Mutants were selfed to obtain stable homozygous lines for phenotyping. DNA was isolated from segregating seedlings and PCR-amplified. Homozygotes were identified by fragment analysis as described by [64]. Analyses were performed on an ABI Prism 3130 Genetic Analyzer using Peak Scanner version 1.0 software. Amplification employed three primers: a specific forward primer (outside the protospacer, Supplementary Table S4) with a 5′ M13 primer tail (50 µM), a reverse primer (also outside the protospacer, 250 µM; Supplementary Table S4), and a universal fluorescent-labeled M13 primer (200 µM). Seedlings homozygous for frameshift mutations were grown and sequence verified before pollination tests.

### 4.5. Pollination Phenotypes

Flowers were emasculated prior to anthesis (−1 stage, [11]) and pollinated a day later (+1 stage) by covering the stigma with *S. lycopersicum* pollen (LA4444 or VF36). After 48 h, 5 to 13 pistils for each cross were prepared and stained with aniline blue fluorochrome (Biosupplies, Melbourne, VIC, Australia) as described [31]. Stained pistils were viewed using a Zeiss Axiovert 200M microscope, and images were processed with MetaMorph v.7.8.12. Style length was measured from the top of the stigma to the base of the style. *S. lycopersicum* pollen tube was assessed using two metrics: the distance from the stigma to the point where only five pollen tubes could be observed (i.e., most pollen tubes stopped) and the distance from the stigma to the tip of the last visible pollen tube (i.e., longest pollen tube). Both metrics were converted to “percent of style traversed” and analyzed separately using the R statistical package (R Development Core Team, 2010). Non-parametric Kruskal–Wallis one-way analysis of variance was used to compare mean pollen tube lengths. Means were compared for candidates having two or three different allelic mutations (*SpAGP3, SpDIR1L, Sp120k, SpTTSR, SpTTSL,* and *SpPEP2*). As no significant differences (*p* < 0.05) were observed between transgenic lines (Supplementary Table S1), pollination data was grouped, and means were used for comparisons with *S. pennellii* LA0716 controls (Supplementary Table S2). Kernel density estimates in Figure 2 were prepared with the R core density function (R Development Core Team, 2010).

### 4.6. Immunoblot Analysis

SpDIR1L antibody was prepared against the peptide SAVSGPKPLPPSDKC (21st Century Biochemicals, Marlboro, MA, USA). Styles were homogenized in 2X SDS–PAGE sample buffer (1 mg fw/10 µL), boiled 5 min, and centrifuged at 21,000× *g* 5 min. Extract equivalent to 1 mg fw was separated in 12.5% Tris/tricine gels [65], blotted to PVDF, and immunostained as described in [22].

### 4.7. Genomic Sequence Mapping

Short read sequence data were downloaded from the European Nucleotide Archive and separately mapped onto the *S. pennellii* LA0716 genome [46]. The region flanking SpDIR1L is shown in Figure 3a. Accessions used include SAMEA2610542 (*S. lycopersicum*, cv. M82), SAMEA2340846 (*S. galapagense*, acc. LA1044), SAMEA2340807 (*S. pimpinellifolium*, acc. LA1584), SAMEA2335233 (*S. arcanum*, acc. LA2157), SAMEA2340818 (*S. arcanum*, acc. LA2172), SAMEA2340815 (*S. neorickii*, acc. LA2133), SAMEA2340833 (*S. huaylasense*, acc. LA1364), SAMEA2340786 (*S. corneliomulleri*, LA0118), SAMEA2340822 (*S. chilense*, acc. CGN15532), SAMEA2340829 (*S. habrochaites*, acc. LA0407), SAMEA2340828 (*S. habrochaites*, acc. LA1777), and SAMEA2340832 (*S. pennellii*, acc. LA0716). Single nucleotide and structural variants between positions 107,160,160 and 107,162,874 of Chr.1 of *S. pennellii* genome (1,200 bp upstream and downstream of *SpDIR1L* start and stop codons, respectively) were called and manually curated. WGS data filtering, alignment, and variant calling was carried out with CLC genomics workbench (v. 10.0.1, CLC bio).

## Figures and Tables

**Table 1:** CRISPR/Cas9-targeted mutations generated in selected candidate pistil-side genes.

Candidate Gene	Expected Position of Mutation/CDS Length (bp)	Transgenic Line	Indel (bp) ^†^	Predicted Change in Protein Sequence ^‡^
*SpAGP3*	157/486	*spagp3-1*	−2	S53I/fs/*59
		*spagp3-2*	−4	S53Q/fs/*88
		*spagp3-3*	−43	P51D/fs/*75
*SpDIR1L*	90/315	*spdir1l-1*	+1	L30F/fs/*37
		*spdir1l -2*	+2	L30F/fs/*34
		*spdir1l -3*	−5	S29V/fs/*35
*Sp120K*	149/1446	*sp120k-1*	−2	G50V/fs/*57
		*sp120k-2*	−7	G52D/fs/*242
		*sp120k-3*	−91	L31A/fs/*216
*SpTTSL*	136/792	*spttsl-1*	−1	P46L/fs/*85
		*spttsl-2*	−8	P46S/fs/*48
		*spttsl-3*	−13	P46L/fs/*81
*SpTTSR*	393/756	*spttsr-1*	−1	K132N/fs/*139
		*spttsr-2*	−2	K132T/fs/*151
*SpPEP2*	112/576	*sppep2-1*	+1	N38K/fs/*42
		*sppep2-2*	−8	P36C/fs/*39
*SpPELPIII*	152/1158	*sppelpIII-1*	+2	D51V/fs/*106
*SpPUR*	80/240	*sppur-1*	−32	M23N/fs/*36

**^†^** “−“: deletion; “+“: insertion. **^‡^** Amino acid change and position/type of mutation (fs, frameshift); * stop codon introduced counting from start codon of the predicted protein.

## Data Availability

Dataset described in ‘4.7. Genomic Sequence Mapping’ was downloaded from the European Nucleotide Archive (https://www.ebi.ac.uk/ena/browser/view, accessed on 2 February 2015).

## Supplementary Materials

The following are available online at https://www.mdpi.com/article/10.3390/ijms222313067/s1.
