# Supplementary material for: A Cysteine-Rich Protein, SpDIR1L, Implicated in S-RNase-Independent Pollen Rejection in the Tomato (Solanum Section Lycopersicon) Clade
Source: Int J Mol Sci. 2021 Dec 2;22(23):13067. doi: 10.3390/ijms222313067 (PMC8657656; doi:10.3390/ijms222313067)
Supplement: Supplementary file 1 [file ijms-22-13067-s001.zip › ijms-1461686-supplementary.pdf]

# Supplementary Materials

(a)

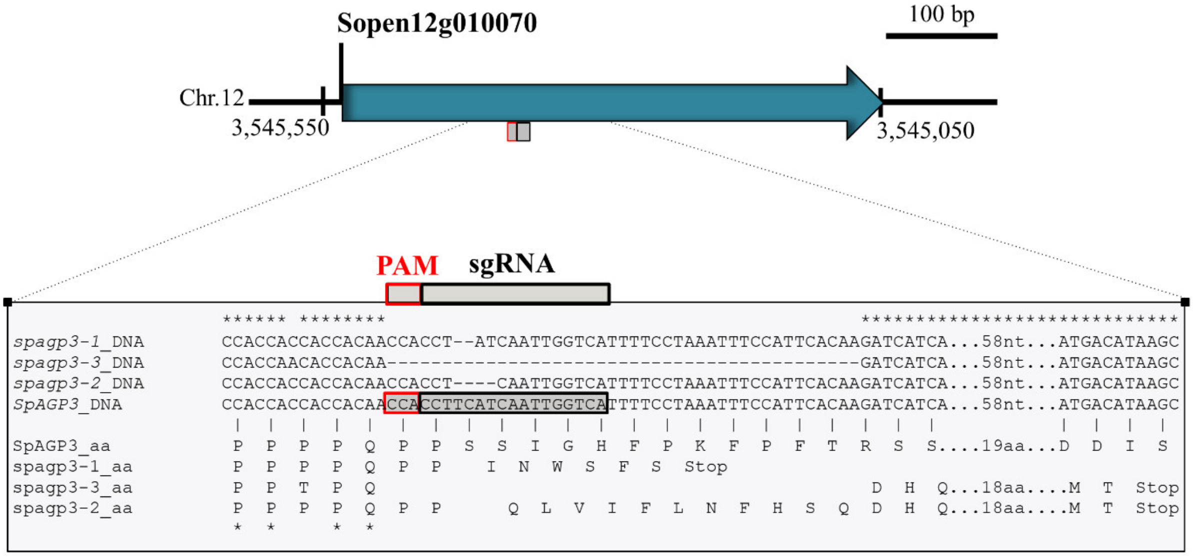


(b)

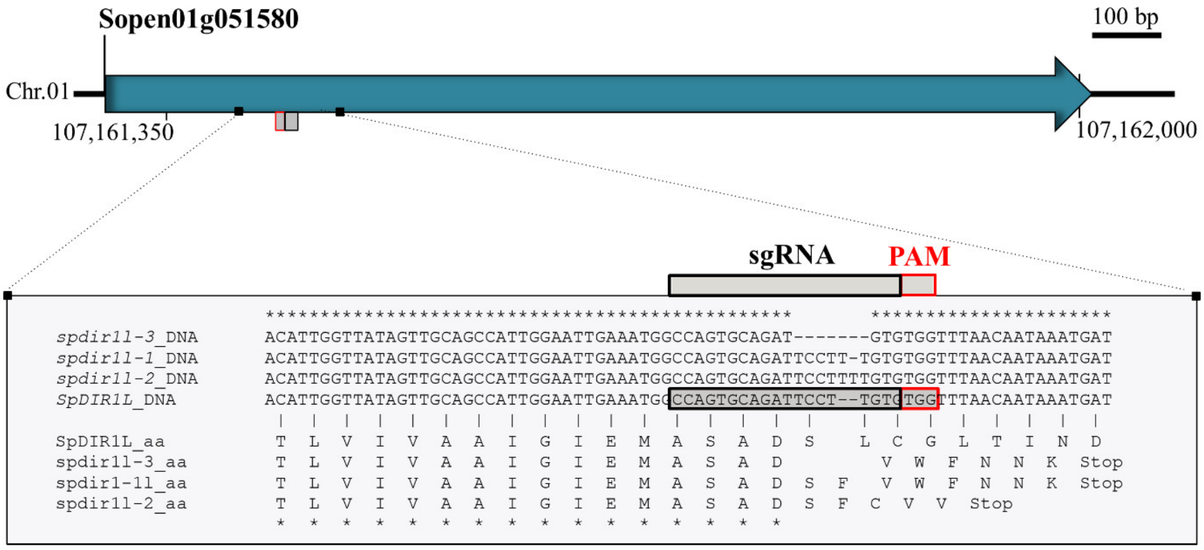

**(c)**

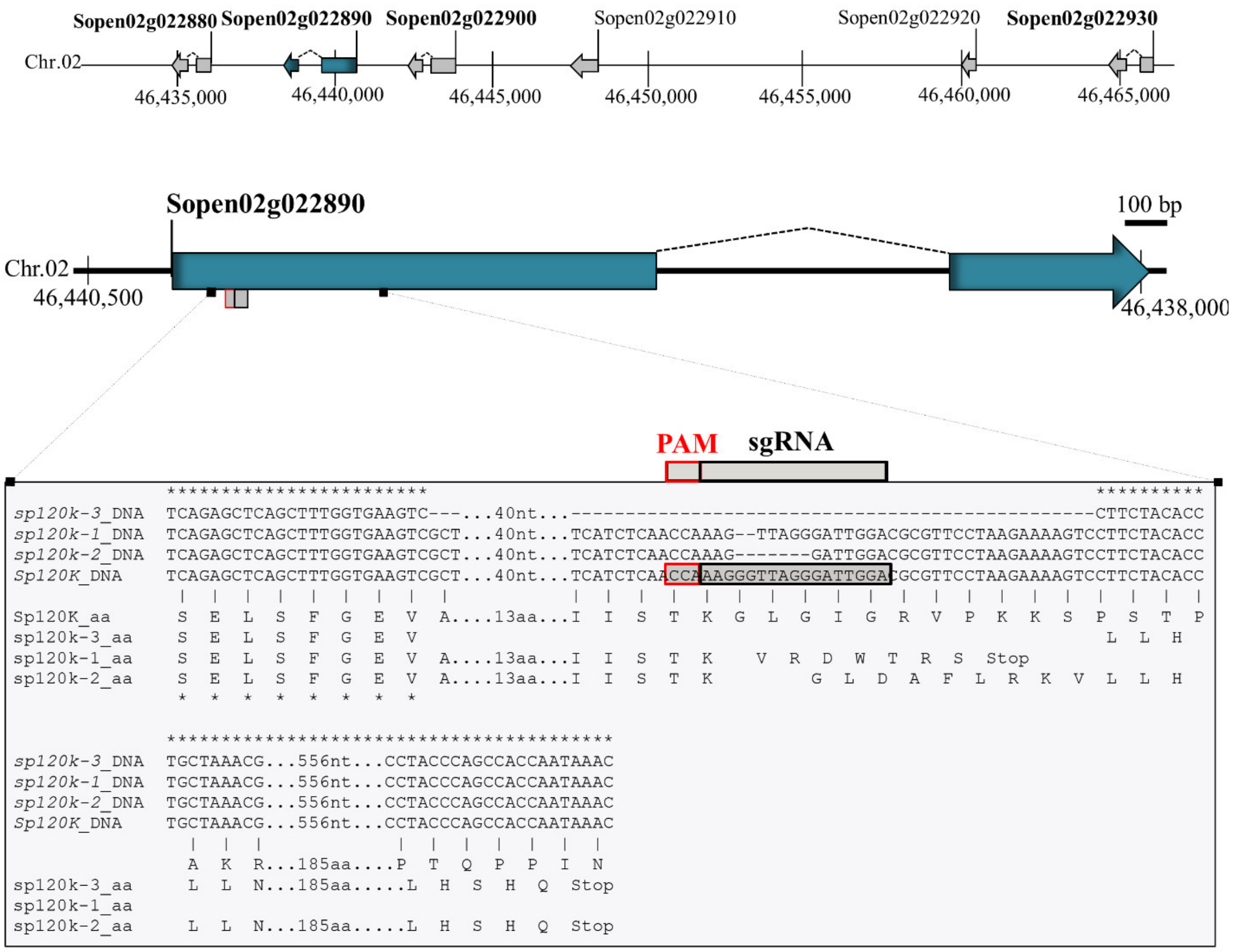

Sopen02g022880
Sopen02g022890
Sopen02g022900
Sopen02g022910
Sopen02g022920
Sopen02g022930
Chr.02
46,435,000
46,440,000
46,445,000
46,450,000
46,455,000
46,460,000
46,465,000
Sopen02g022890
100 bp
Chr.02
46,440,500
46,438,000
PAM
sgRNA
sp120k-3_DNA TCAGAGCTCAGCTTTGGTGAAGTC---...40nt...-------------------------------------------------CTTCTACACC
sp120k-1_DNA TCAGAGCTCAGCTTTGGTGAAGTCGCT...40nt...TCATCTCAACCAAAG--TTAGGGATTGGACGCGTTCCTAAGAAAAGTCCTTCTACACC
sp120k-2_DNA TCAGAGCTCAGCTTTGGTGAAGTCGCT...40nt...TCATCTCAACCAAAG-------GATTGGACGCGTTCCTAAGAAAAGTCCTTCTACACC
Sp120K_DNA TCAGAGCTCAGCTTTGGTGAAGTCGCT...40nt...TCATCTCAACCAAAGGGTTAGGGATTGGACGCGTTCCTAAGAAAAGTCCTTCTACACC
Sp120K_aa S E L S F G E V A....13aa...I I S T K G L G I G R V P K K S P S T P
sp120k-3_aa S E L S F G E V L L H
sp120k-1_aa S E L S F G E V A....13aa...I I S T K V R D W T R S Stop
sp120k-2_aa S E L S F G E V A....13aa...I I S T K G L D A F L R K V L L H
sp120k-3_DNA TGCTAAACG...556nt...CCTACCCAGCCACCAATAAAC
sp120k-1_DNA TGCTAAACG...556nt...CCTACCCAGCCACCAATAAAC
sp120k-2_DNA TGCTAAACG...556nt...CCTACCCAGCCACCAATAAAC
Sp120K_DNA TGCTAAACG...556nt...CCTACCCAGCCACCAATAAAC
A K R...185aa....P T Q P P I N
sp120k-3_aa L L N...185aa.....L H S H Q Stop
sp120k-1_aa
sp120k-2_aa L L N...185aa.....L H S H Q Stop


**(d)**

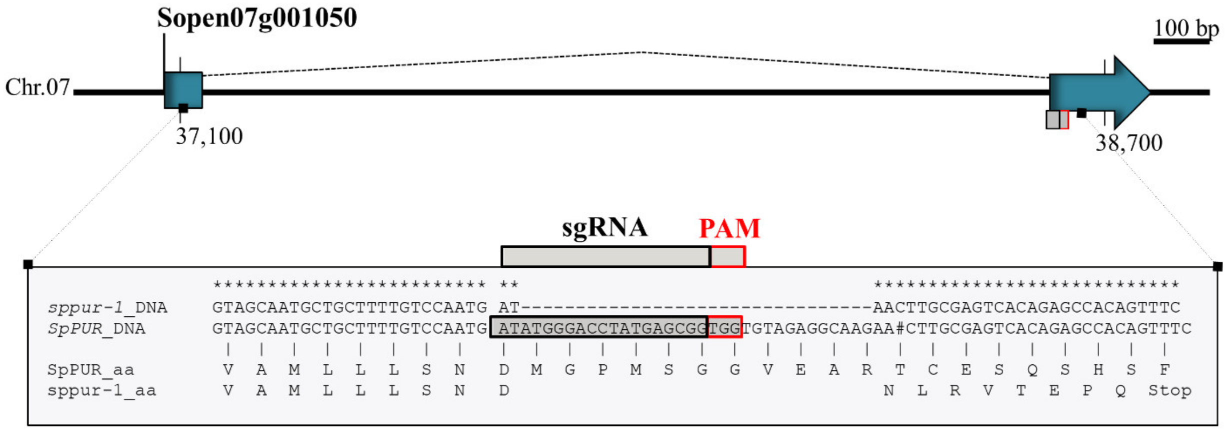

Sopen07g001050
100 bp
Chr.07
37,100
38,700
sgRNA
PAM
sppur-1_DNA GTAGCAATGCTGCTTTTGTCCAATG AT-------------------------------AACTTGCGAGTCACAGAGCCACAGTTTC
SpPUR_DNA GTAGCAATGCTGCTTTTGTCCAATG ATATGGGACCTATGAGCGGTGGTGTAGAGGCAAGAA#CTTGCGAGTCACAGAGCCACAGTTTC
SpPUR_aa V A M L L L S N D M G P M S G G V E A R T C E S Q S H S F
sppur-1_aa V A M L L L S N D N L R V T E P Q Stop

**(e)**

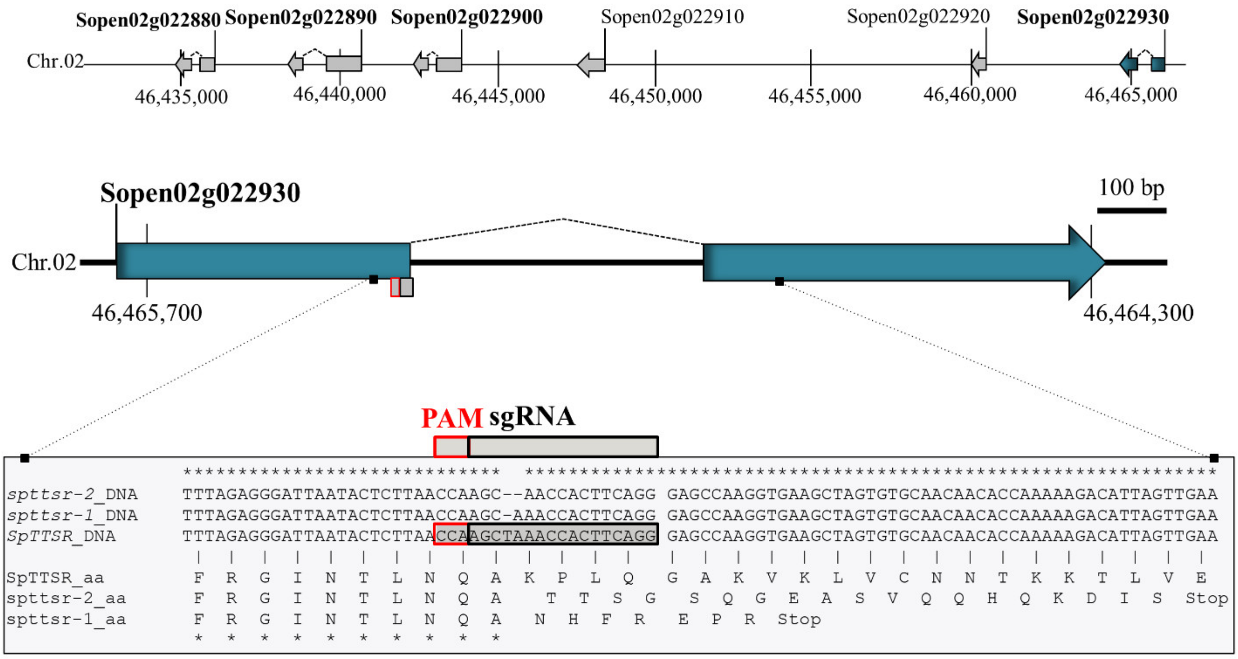
Sopen02g022880
Sopen02g022890
Sopen02g022900
Sopen02g022910
Sopen02g022920
Sopen02g022930
Chr.02
46,435,000
46,440,000
46,445,000
46,450,000
46,455,000
46,460,000
46,465,000
Sopen02g022930
100 bp
Chr.02
46,465,700
46,464,300
PAM
sgRNA
spttsr-2_DNA TTTAGAGGGATTAATACTCTTAACCAAGC--AACCACTTCAGG GAGCCAAGGTGAAGCTAGTGTGCAACAACACCAAAAAGACATTAGTTGAA
spttsr-1_DNA TTTAGAGGGATTAATACTCTTAACCAAGC-AAACCACTTCAGG GAGCCAAGGTGAAGCTAGTGTGCAACAACACCAAAAAGACATTAGTTGAA
SpTTSR_DNA TTTAGAGGGATTAATACTCTTAACCAAGCTAAACCACTTCAGG GAGCCAAGGTGAAGCTAGTGTGCAACAACACCAAAAAGACATTAGTTGAA
SpTTSR_aa F R G I N T L N Q A K P L Q G A K V K L V C N N T K K T L V E
spttsr-2_aa F R G I N T L N Q A T T S G S Q G E A S V Q Q H Q K D I S Stop
spttsr-1_aa F R G I N T L N Q A N H F R E P R Stop

**(f)**

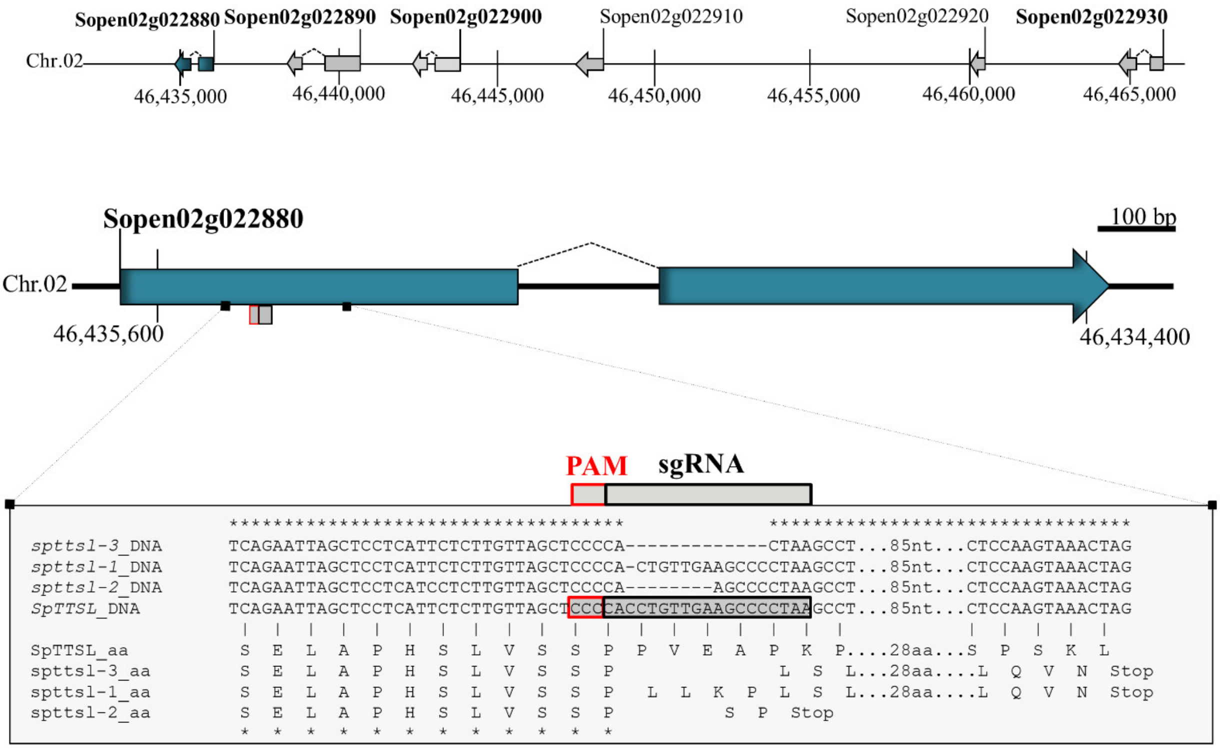
Sopen02g022880
Sopen02g022890
Sopen02g022900
Sopen02g022910
Sopen02g022920
Sopen02g022930
Chr.02
46,435,000
46,440,000
46,445,000
46,450,000
46,455,000
46,460,000
46,465,000
Sopen02g022880
100 bp
Chr.02
46,435,600
46,434,400
PAM
sgRNA
spttsl-3_DNA TCAGAATTAGCTCCTCATTCTCTTGTTAGCTCCCCA-------------CTAAGCCT...85nt...CTCCAAGTAAACTAG
spttsl-1_DNA TCAGAATTAGCTCCTCATTCTCTTGTTAGCTCCCCA-CTGTTGAAGCCCCTAAGCCT...85nt...CTCCAAGTAAACTAG
spttsl-2_DNA TCAGAATTAGCTCCTCATCCTCTTGTTAGCTCCCCA--------AGCCCCTAAGCCT...85nt...CTCCAAGTAAACTAG
SpTTSL_DNA TCAGAATTAGCTCCTCATTCTCTTGTTAGCTCCCCACCTGTTGAAGCCCCTAAGCCT...85nt...CTCCAAGTAAACTAG
SpTTSL_aa S E L A P H S L V S S P P V E A P K P....28aa...S P S K L
spttsl-3_aa S E L A P H S L V S S P L S L...28aa....L Q V N Stop
spttsl-1_aa S E L A P H S L V S S P L L K P L S L...28aa....L Q V N Stop
spttsl-2_aa S E L A P H S L V S S P S P Stop

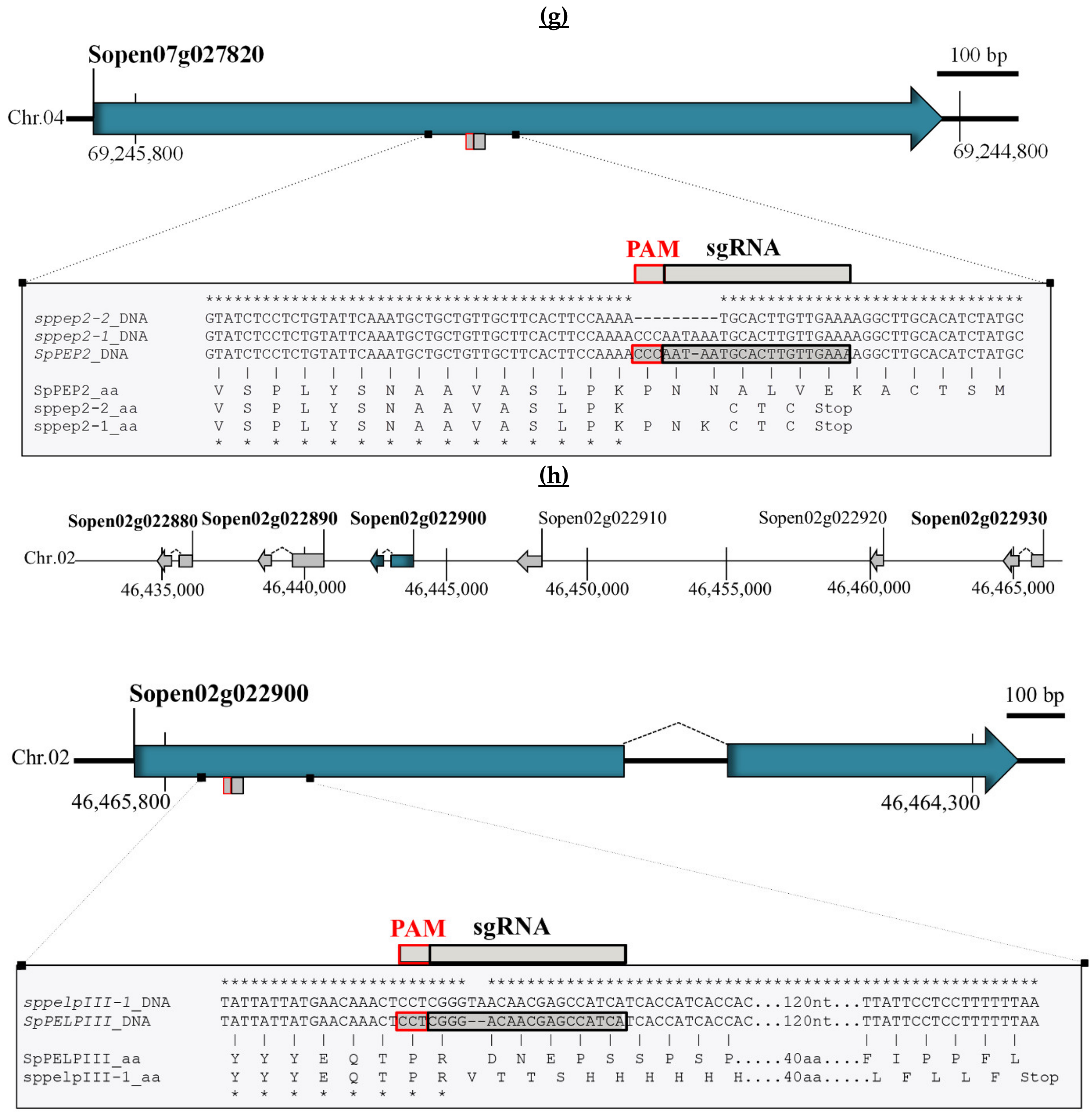


**Figure S1. Loss of function mutations.** Each panel shows the relevant *S. pennellii* LA0716 gene model including sequence coordinates, exons (blue arrows), and a 100bp scale bar. Boxes below show the CRISPR/Cas9 target, sgRNA and PAM sequences. Relevant sequences of the wild type and loss of function mutations are shown below. Asterisks, identical nucleotides or amino acids. Premature termination codons are indicated as "Stop". Splicing site, space in the sequence. (**a**) *SpAGP3*, (**b**) *SpDIR1L*, (**c**) *Sp120k*, (**d**), *SpPUR* (**e**) *SpTTSR*, (**f**) *SpTTSL*, (**g**) *SpPEP2* and (**h**) *SpPELPIII*. For (c), (d), (e) and (f) the chromosome 2 region including all four genes is shown.

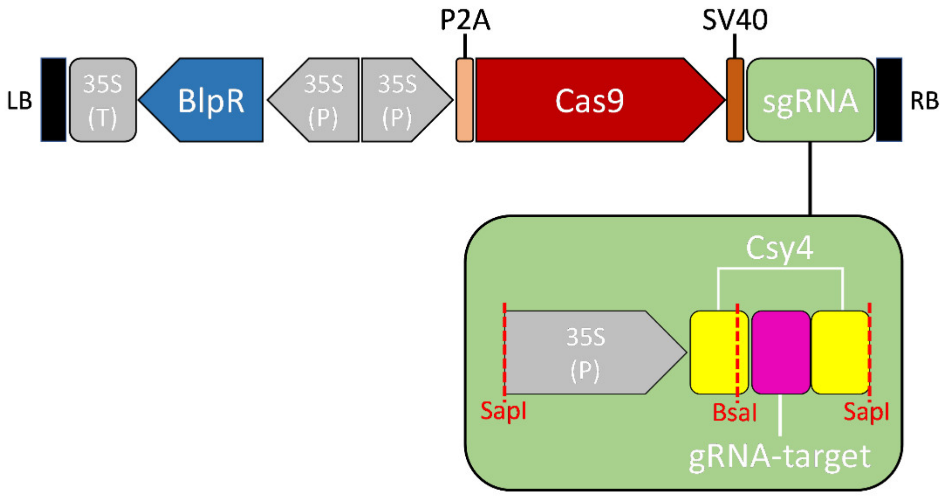


**Figure S2. Schematic of pRLG108 CRISPR/Cas9 constructs.** The sgRNA encoding region is enlarged showing Cys4 cleavage sites and BsaI-SapI sites for gene-specific inserts (Table S3).

**Table S1.** *Solanum lycopersicm* pollen tube growth in styles of LA0716 transgenic lines and wild type.

| Gene | LOF allele | Style length (mm) | Most metric (% of style) | Longest metric (% of style) |
|---|---|---|---|---|
| **LA0716** | | 7.17±0.76 | 1.72±0.51 (23.98±7.79%) | 2.02±0.53 (28.17±8.00%) |
| ***SpPEP2*** | *sppep2-1* | 8.34±0.51 | 1.45±0.84 (17.38±9.05%) | 1.55±0.93 (18.58±10.05%) |
| | *sppep2-2* | 6.98±0.23 | 0.86±0.36 (12.32±5.38%) | 1.11±0.49 (15.9±7.40%) |
| ***SpTTSL*** | *spttsl-1* | 8.91±0.90 | 1.87±0.56 (20.98±6.92%) | 2.14±0.37 (24.01±5.36%) |
| | *spttsl-2* | 6.93±0.70 | 1.62±0.33 (23.37±5.06%) | 1.89±0.53 (27.27±8.53%) |
| | *spttsl-3* | 7.41±0.54 | 1.24±0.57 (16.73±7.07%) | 2.02±0.74 (27.26±9.81%) |
| ***SpTTSR*** | *spttsr-1* | 6.76±0.43 | 1.22±0.52 (18.04±7.15%) | 1.73±0.72 (25.59±9.66%) |
| | *spttsr-2* | 8.07±0.27 | 1.99±0.45 (24.65±5.97%) | 2.42±0.64 (29.98±8.48%) |
| ***SpAGP3*** | *agp3-1* | 6.61±0.18 | 1.61±0.38 (24.35±7.83%) | 1.94±0.47 (29.34±6.71%) |
| | *agp3-2* | 7.61±0.51 | 1.53±0.36 (20.1±4.45%) | 2.72±0.35 (35.74±6.27%) |
| | *agp3-3* | 6.97±0.44 | 1.58±0.33 (22.66±5.71%) | 2.09±0.39 (29.98±6.13%) |
| ***Sp120k*** | *Sp120k-1* | 6.61±0.45 | 1.76±0.53 (26.62±8.61%) | 2.13±0.47 (32.22±7.64%) |
| | *Sp120k-2* | 7.13±0.42 | 2.06±0.44 (29.51±7.14%) | 2.21±0.65 (30.99±10.13%) |
| | *Sp120k-3* | 7.86±0.87 | 2.32±0.63 (29.51±6.92%) | 2.66±0.24 (33.84±2.61%) |
| ***SpPELPIII*** | *sppelpIII-1* | 7.54±0.79 | 2.22±0.64 (29.44±9.85%) | 2.44±0.53 (32.36±8.34%) |
| ***SpPur*** | *sppur-1* | 6.9±0.36 | 2.16±0.59 (31.3±8.67%) | 3.03±1.06 (43.91±16.01%) |
| ***SpDIR1L*** | *spdir1l-1* | 7.39±0.71 | 2.53±0.68 (34.23±9.91%) | 3.27±1.17 (44.24±15.77%) |
| | *spdir1l-2* | 6.94±0.83 | 3.24±0.98 (46.68±15.16%) | 3.96±1.34 (57.06±21.13%) |
| | *spdir1l-3* | 7.22±0.76 | 2.8±1.05 (38.78±14.15%) | 4.01±1.88 (55.54±23.77%) |

(a) Mean and standard deviation of most and longest pollen tube metrics for loss of function (LOF) mutants and controls (yellow).

| Gene | df | Most metric | | | Longest metric | | |
|---|---|---|---|---|---|---|---|
| | | Mean±SD | chi-sq | p-value | Mean±SD | chi-sq | p-value |
| ***SpPEP2*** | 1 | 14.7±7.5% | 0.641 | 0.423 | 17.1±8.5% | 0.025 | 0.872 |
| ***SpTTSL*** | 2 | 19.7±7.0% | 4.35 | 0.113 | 26.6±8.3% | 0.557 | 0.756 |
| ***SpTTSR*** | 1 | 22.0±7.1% | 2.907 | 0.088 | 25.1±8.9% | 1.114 | 0.291 |
| ***SpAGP3*** | 2 | 22.5±5.9% | 0.929 | 0.628 | 28.8±6.5% | 1.71 | 0.425 |
| ***Sp120k*** | 2 | 28.4±7.5% | 2.009 | 0.366 | 32.4±7.8% | 2.664 | 0.263 |
| ***SpPELPIII*** | - | 29.9±9.8% | - | - | 32.8±8.3% | - | - |
| ***SpPUR*** | - | 31.4±8.7% | - | - | 44.2±16.0% | - | - |
| ***SpDIR1L*** | 2 | 41.6±14.4% | 4.215 | 0.121 | 54.2±21.1% | 1.354 | 0.508 |

(b) Effects of *S. lycopersicum* pollen tube rejection on different alleles (where available) of the same candidate. Separate Kruskal–Wallis one-way analysis of variance to tests were applied to each metric. Tests show no significant difference between alleles supporting pooling data in Figure 2.

**Table S2.** Kruskal-Wallis one-way analysis of variance to tests comparing loss of function mutations to control. P-values for most and longest metrics are shown.

| Candidate gene knock-out | P-values | |
|---|---|---|
| | Most of pollen tubes | Longest pollen tubes |
| *sppep2* | 0.003* | 0.005* |
| *spttsl* | 0.072 | 0.491 |
| *spttsrr* | 0.513 | 0.913 |
| *tagp3* | 0.292 | 0.792 |
| *sp120k* | 0.153 | 0.168 |
| *sppelpIII-1* | 0.246 | 0.277 |
| *sppur-1* | 0.059 | 0.002* |
| *spdir1l* | <0.001** | <0.001** |

* Significant differences (P<0.05). ** Significant differences (P<0.001).

**Table S3.** Sequences synthesized for CRISPR/Cas9 constructs. Specific gRNA gene-targeting sequences are bold and highlighted in pink. Yellow, Csy4 cleavage targets. Grey, protospacer. Blue, 5′ BsaI sites; green, 3′ SapI sites.

| Candidate gene | DNA-synthesized fragment |
|---|---|
| *SpAGP3* | cgTGGTCTCAGCAG**ATGACCAATTGATGAAGG**GTTTTAGAGCTAGAAATAGCAAGTTAAAA-<br>TAAGGCTAGTCCGTTATCAACTTGAAAAAGTGGCACCGAGTCGGTGCGTTCACTGCCGTATAGGCAGGT<br>CAGAAGAGCATG |
| *Sp120k* | cgTGGTCTCAGCAG**GTCCAATCCCTAACCCTT**GTTTTAGAGCTAGAAATAGCAAGTTAAAA-<br>TAAGGCTAGTCCGTTATCAACTTGAAAAAGTGGCACCGAGTCGGTGCGTTCACTGCCGTATAGGCAGGT<br>CAGAAGAGCATG |
| *SpTTSR* | cgTGGTCTCAGCAG**CCCTGAAGTGGTTTAGCT**GTTTTAGAGCTAGAAATAGCAAGTTAAAA-<br>TAAGGCTAGTCCGTTATCAACTTGAAAAAGTGGCACCGAGTCGGTGCGTTCACTGCCGTATAGGCAGGT<br>CAGAAGAGCATG |
| *SpPELPIII* | cgTGGTCTCAGCAG**TGATGGCTCGTTGTCCCG**GTTTTAGAGCTAGAAATAGCAAGTTAAAA-<br>TAAGGCTAGTCCGTTATCAACTTGAAAAAGTGGCACCGAGTCGGTGCGTTCACTGCCGTATAGGCAGGT<br>CAGAAGAGCATG |
| *SpTTSL* | cgTGGTCTCAGCAG**TAGGGGCTTCAACAGGTG**GTTTTAGAGCTAGAAATAGCAAGTTAAAA-<br>TAAGGCTAGTCCGTTATCAACTTGAAAAAGTGGCACCGAGTCGGTGCGTTCACTGCCGTATAGGCAGGT<br>CAGAAGAGCATG |
| *SpPEP2* | cgTGGTCTCAGCAG**AGAGGAGATACACAGAAC**GTTTTAGAGCTAGAAATAGCAAGTTAAAA-<br>TAAGGCTAGTCCGTTATCAACTTGAAAAAGTGGCACCGAGTCGGTGCGTTCACTGCCGTATAGGCAGGT<br>CAGAAGAGCATG |

**Table S4.** Primers used to identify and validate CRISPR/Cas9-induced mutations.

| Candidate gene | Primer name† | Sequence |
|---|---|---|
| ***SpAGP3*** | SpAGP3-OutF | CGTTCTGACTGTTGATCATGCTTATGT |
| | SpAGP3-FlaF | GAATGGAAATTTAGGAAAATGACCAATTGATGAA |
| | SpAGP3-OutR | GAGGTTATAGGAGTGATTGTTTTGTGAGG |
| ***Sp120k*** | Sp120K-outF | GTGAAGGTGGTGGTGGTGATTG |
| | Sp120k-FlaF | ACGCGTCCAATCCCTAACCC |
| | Sp120k-OutR | CAAAGGCCATGGTGCTCATACA |
| ***SpTTSR*** | SpTTSR-OutF | GGACATTTGAAGCACGGACACTATAAG |
| | SpTTSR-FlaF | AAGAAGAAATTACCCTGAAGTGGTTTAG |
| | SpTTSR-OutR | CAGCTAAGCCACCATCACCTTATTATC |
| ***SpPELPIII*** | SpPELPIII-OutF | GTGGCGTCGTTGGGTTAATAGT |
| | SpPELPIII-FlaF | GGTGATGATGGCTCGTTGTCC |
| | SpPELPIII-OutR | GCAGGGAAAGGCGTGCTAATAC |
| ***SpTTSL*** | SpTTSL-OutF | AACTTTCTGACCGGAAGAGGAGA |
| | SpTTSL-FlaF | AGGCTTAGGGGCTTCAACAGG |
| | SpTTSL-OutR | TTCCAACTAAGTCCCTTGGGTTTATTC |
| ***SpPEP2*** | SpPEP2-OutF | GCCTGAATTGCTTCTGTGCTCT |
| | SpPEP2-FlaF | TGCAAGCCTTTTCAACAAGTGCATTA |
| | SpPEP2-OutR | GAGCAAAGGCCAAGTCGAAAATG |

| | | |
|---|---|---|
| ***SpDIR1L*** | SpDIR1L-OutF | CAACAACTAGCCAAGGTCTTCATAATAATCC |
| | SpDIR1L-FlaF | GAAATGGCCAGTGCAGATTCCTTG |
| | SpDIR1L-OutR | AAGTGCAAAGGCAAGGCAAATC |
| ***SpPUR*** | SpPUR-OutF | TGCCTTATTTATCAAAGGCAAGAGCC |
| | SpPUR-FlaF | TAATTGATAGATATGGGACCTATGAGC |
| | SpPUR-OutR | CTACTACATGGCCCCTTGAAACTGTG |

† OutF, forward primers outside the protospacer. FlaF, forward primers flanking protospacer. OutR, reverse primers outside the protospacer.
